# NSUN2 promotes colorectal cancer progression and increases lapatinib sensitivity by enhancing CUL4B/ErbB‐STAT3 signalling in a non‐m5C manner

**DOI:** 10.1002/ctm2.70282

**Published:** 2025-03-28

**Authors:** Yuanbo Hu, Chenbin Chen, Kezhi Lin, Xinya Tong, Tingting Huang, Tianle Qiu, Xietao Chen, Jun Xu, Wangkai Xie, Xiangwei Sun, Shiyu Feng, Mingdong Lu, Zhiguang Zhao, Xiaodong Chen, Xiangyang Xue, Xian Shen

**Affiliations:** ^1^ Department of General Surgery The First Affiliated Hospital of Wenzhou Medical University Wenzhou China; ^2^ Department of General Surgery The Second Affiliated Hospital and Yuying Children's Hospital of Wenzhou Medical University Wenzhou China; ^3^ Zhejiang Key Laboratory of Intelligent Cancer Biomarker Discovery and Translation The First Affiliated Hospital of Wenzhou Medical University Wenzhou China; ^4^ Department of Microbiology and Immunology, Institute of Molecular Virology and Immunology, School of Basic Medical Sciences Wenzhou Medical University Wenzhou China; ^5^ Experiemtial Center of Basic Medicine, School of Basic Medical Sciences Wenzhou Medical University Wenzhou China; ^6^ Department of Obstetrics and Gynecology, Maternal and Child Care Service Hospital Yueqing China; ^7^ Department of Pathology The Second Affiliated Hospital & Yuying Children's Hospital of Wenzhou Medical University Wenzhou China

**Keywords:** Colorectal cancer, ErbB‐STAT3 signalling pathway, m5C‐independent function, NSUN2

## Abstract

NSUN2, a major methyltransferase that catalyzes m5C methylation in eukaryotes, is known to be implicated in the development of multiple cancers. However, its role in colorectal cancer (CRC) and the related molecular mechanisms have yet to be sufficiently determined. Here, we conducted an analysis of public database (722 CRC patients) and two distinct cohorts from our centre (1559 CRC patients), which revealed that NSUN2 is upregulated in CRC and correlates with unfavourable prognosis. Our analyses also showed that NSUN2 promotes the proliferation and metastasis capabilities of CRC cells. Intriguingly, NSUN2 was found to promote CRC via an m5C‐independent mechanism, which has not been previously reported. Overexpression of both wild‐type and m5C enzymatic‐dead mutant NSUN2 upregulated and activated the ErbB‐STAT3 signalling pathway. We also found that both wild‐type and the m5C enzymatic‐dead mutant NSUN2 closely interacted with CUL4B. Silencing of CUL4B effectively inhibited the m5C‐independent function of NSUN2. Moreover, overexpression of NSUN2 enhanced the sensitivity of CRC cells to lapatinib. Taken together, our findings revealed a novel m5C‐independent mechanism for NSUN2 in the malignancy and lapatinib sensitivity of CRC via activation of the CUL4B/ErbB‐STAT3 pathway, which provides a potential therapeutic strategy for patients with CRC.

**Highlights:**

- NSUN2 is upregulated in CRC and associated with poor prognosis of CRC patients.
- NSUN2 promotes CRC malignancy independently of its m5C‐enzymatic activity, a mechanism that has not been previously reported.
- The non‐m5C carcinogenic roles of NSUN2 may be mediated through interactions with CUL4B, thereby activating the ErbB‐STAT3 signalling pathway.
- NSUN2‐mediated upregulation of ErbB‐STAT3 pathway enhances the sensitivity of CRC to lapatinib treatment.

## Graphical abstract

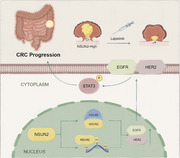

## 1 INTRODUCTION

Colorectal cancer (CRC) is the third most common malignancy and the second leading cause of cancer‐related mortality worldwide.^1^, ^2^ The pathogenesis of CRC remains unclear, although various contributing factors have been identified, including genetics, obesity and environmental factors. Common therapeutic approaches for CRC include surgery, chemotherapy and targeted immunotherapies.^3^ However, despite advancements in therapeutic strategies, the 5‐year survival rate of patients with advanced metastatic CRC remains only 14%.^4^, ^5^ Therefore, it is imperative to investigate the mechanisms underlying the pathogenesis of CRC and identify novel biomarkers to develop new treatment strategies.

Non‐mutational epigenetic reprogramming, such as RNA modifications, is a hallmark of malignancy.^6^ Accumulating evidence has shown that these RNA modifications, especially N6‐methyladenosine (m6A), have multi‐biological effects.^7^, ^8^ The 5‐methylcytosine (m5C) modification has recently attracted attention and participated in the tumourigenesis of various human cancers, including gastric cancer,^9^ lung cancer,^10^ hepatocellular carcinoma^11^ and prostate cancer.^12^ Dysregulation of RNA modification in tumours is primarily driven by changes in the expression of regulators that modulate the expression of downstream genes.^13^ The m5C methyltransferases in eukaryotes are DNMT2 and NSUN1–7.^14^, ^15^ NSUN2 is the primary catalyst of m5C modifications in messenger RNA (mRNA).^16^ A wealth of research indicates that NSUN2 was highly expressed in a variety of cancers and functions to regulate downstream mRNA expression, stability or translation in a m5C‐dependent manner, and ultimately promoting malignant progression. However, NSUN2 was initially identified for its role in maintaining spindle stability during mitosis as part of an RNA–protein complex, which implies that some of the functions of NSUN2 in tumours may not depend on m5C modification. Recently, the methyltransferase‐independent functions of RNA modification regulators have attracted increasing attention. For example, the typical m6A methyltransferase complex METTL3/METTL14 has been reported to play a key role in maintaining nucleolar phase separation, and this function is independent of its m6A catalytic activity.^17^ The m6A methyltransferase METTL6 exerts m6A writer function in the nucleus and interacts with EIF3a/b in an m6A‐independent manner to promote translation in the cytosol.^18^ As gastrointestinal cancers (gastric cancer and CRC) are exposed to a dynamic internal environment replete with physiological stimuli, a multitude of complex factors, such as nutrients, pharmaceuticals and pathogenic microorganisms, may collectively contribute to regulating the initiation and progression of tumours. Consequently, oncogenes and even drug therapy may play more complex regulatory roles in gastrointestinal tumours.^19^ In our previous study, we discovered that NSUN2 contributes to the progression of gastric cancer via both m5C‐dependent and m5C‐independent pathways.^9^ However, the putative m5C‐independent biological functions of NSUN2 in CRC remain to be elucidated. Therefore, it is important to explore the m5C‐independent functions of NSUN2 in tumour progression, with the aim of identifying targeted therapeutic approaches for the treatment of CRC that focus on NSUN2 as a potential therapeutic target.

Here, we confirmed the high expression of NSUN2 in CRC using both public datasets (TCGA and GEO databases, containing 722 patients) and two distinct cohorts from our centre (266 patients in Cohort 1 and 1299 patients in Cohort 2) and its correlation with poor prognosis. Subsequently, we verified that NSUN2 facilitated the proliferation and metastatic abilities of CRC cells both in vitro and in animal models. Additionally, we found that an enzymatically inactive NSUN2 mutant can still promote CRC progression. Mechanistically, we discovered that NSUN2, independent of its m5C enzymatic activity, interacted with Cullin‐4B (CUL4B) to activate the EGFR/HER2‐STAT3 pathway, thereby promoting the malignant phenotype of CRC. Moreover, high NSUN2 expression enhanced the sensitivity of CRC cells to lapatinib. In summary, our research presents the m5C‐independent biological functions of NSUN2 in CRC and suggests that patients with CRC expressing high levels of NSUN2 could be treated with lapatinib.

## 2 MATERIALS AND METHODS

### 2.1 Patients and tissue samples

A total of 300 patients in the First Affiliated Hospital of Wenzhou Medical University (January 2014 and December 2016, follow‐up to December 2022) and 1500 patients in the Second Affiliated Hospital of Wenzhou Medical University (January 2018 and April 2023, follow‐up to June 2024), who underwent surgical resection for CRC, were included in this study as Cohort1 and Cohort2, respectively. All the CRC tissues and paired adjacent normal tissues (at least 10 cm away from the negative margin) were formalin fixed and paraffin embedded to construct CRC tissue microarrays, and ultimately confirmed by histopathologic analysis. Clinical survival characteristics were collected for each patient. After immunohistochemistry (IHC) staining, the spots with more than 50% tissue loss were excluded, and finally 266 CRC tissues and 26 adjacent normal tissues were left in the Cohort1 and 1293 CRC samples in the Cohort2. The study was approved by the Review Board of the First Affiliated Hospital of Wenzhou Medical University (KY2022‐202) and Second Affiliated Hospital of Wenzhou Medical University (2021‐K‐42‐01). Written informed consent was obtained from the patients before the study began.

### 2.2 RNA m5C dot blot assay

Total RNA was extracted from CRC cells, and then treated with deoxyribonuclease I (DNase) and quantified for the purpose of loading varying RNA amounts (400, 600, 800 ng) onto Hybond‐N+ membranes (Beyotime). Post a rapid drying phase, the membranes underwent UV crosslinking at 254 nm for 60 s, followed by blocking with 5% milk for 1 h and subsequent overnight incubation at 4°C with an anti‐m5C antibody and corresponding secondary antibody. Ultimately, the membranes were analyzed using enhanced chemiluminescence and visualized with the Bio‐Rad Imaging System.

### 2.3 Xenograft and lung metastasis models

For xenograft studies, female BALB/c nude mice (4 weeks) were randomly assigned to one of two groups. CRC cells overexpressing NSUN2, NSUN2 knockout cells and control cells were harvested (2 × 10^6^) and subcutaneously implanted into the flank regions of female BALB/c nude mice (4 weeks). Subsequently, the volumes of developing tumours were estimated based on length and width measurements (length × width^2^ × .5) obtained at 2‐day intervals. When tumours had reached a predetermined size, the mice were euthanized, and tumour specimens were excised. Finally, xenografts samples were thereafter sectioned at 5 µm for subsequent immunohistochemical analysis.

For lung metastasis models, NSUN2 overexpressing CRC cells were transfected with lentivirus containing a luciferase reporter gene (OBiO Technology). To generate a lung metastasis model, mice were randomly assigned to one of two groups of nude mice, and we injected CRC cells (5 × 10^5^) and intraperitoneally administered d‐luciferin (15 mg/kg, BioGold) into the tail veins. At 5‐day intervals, the mice were placed under isoflurane‐induced anaesthesia and imaged for luciferin emission using an In Vivo Imaging System (IVIS) Spectrum imaging system (PerkinElmer) to track cancer cell dissemination.

All animals used in this study were purchased from Hangzhou Medical College. All animal experiments were performed in compliance with the Wenzhou University's Policy on the Care and Use of Laboratory Animals (WZU‐2024‐074/075).

### 2.4 Lapatinib sensitivity assay

For lapatinib IC50 assays, HCT116 and DLD‐1 cells were plated in 96‐well plates (5000 cells/well) and transfected with either wild‐type NSUN2 (NSUN2‐WT) or double mutant NSUN2 (NSUN2‐DM) plasmids. Following treatment with a gradient of lapatinib (MCE) concentrations, cells were incubated for 48 h before determining the IC50 using CCK‐8 assay.

For in vitro assays, mice were subcutaneously injected with DLD‐1 cells overexpressing NSUN2 (OE) or control cells (NC); two groups received daily oral gavage of lapatinib at a concentration of 50 mg/kg. Subcutaneous tumour volumes were subsequently recorded, and when tumours had reached a predetermined size, the mice were euthanized, followed by the excision and documentation of tumour tissues. The tissues were paraffin‐embedded and sectioned to 5 µm for subsequent analysis.

### 2.5 Statistical analysis

PASW Statistics (version 18; IBM), GraphPad Prism 7.0 software, and R program (version 4.2.3) were used for Statistical analysis. Data are presented as the means ± SD or percentage. Statistical comparisons between the two groups were conducted utilizing a two‐tailed Student's *t*‐test or χ^2^ test. Kaplan–Meier analysis was employed for survival analysis, and the log‐rank test was utilized to compare survival curves. Multivariable Cox regression analysis was performed to exclude confounding factors affecting survival outcomes. The correlation between the expression levels of two genes was evaluated using Spearman correlation analysis. Note that *p < .05, **p < .01 and ***p < .001 were considered statistically significant.

## 3 RESULTS

### 3.1 NSUN2 is highly expressed in CRC and predicts poor prognosis

**FIGURE 1:**
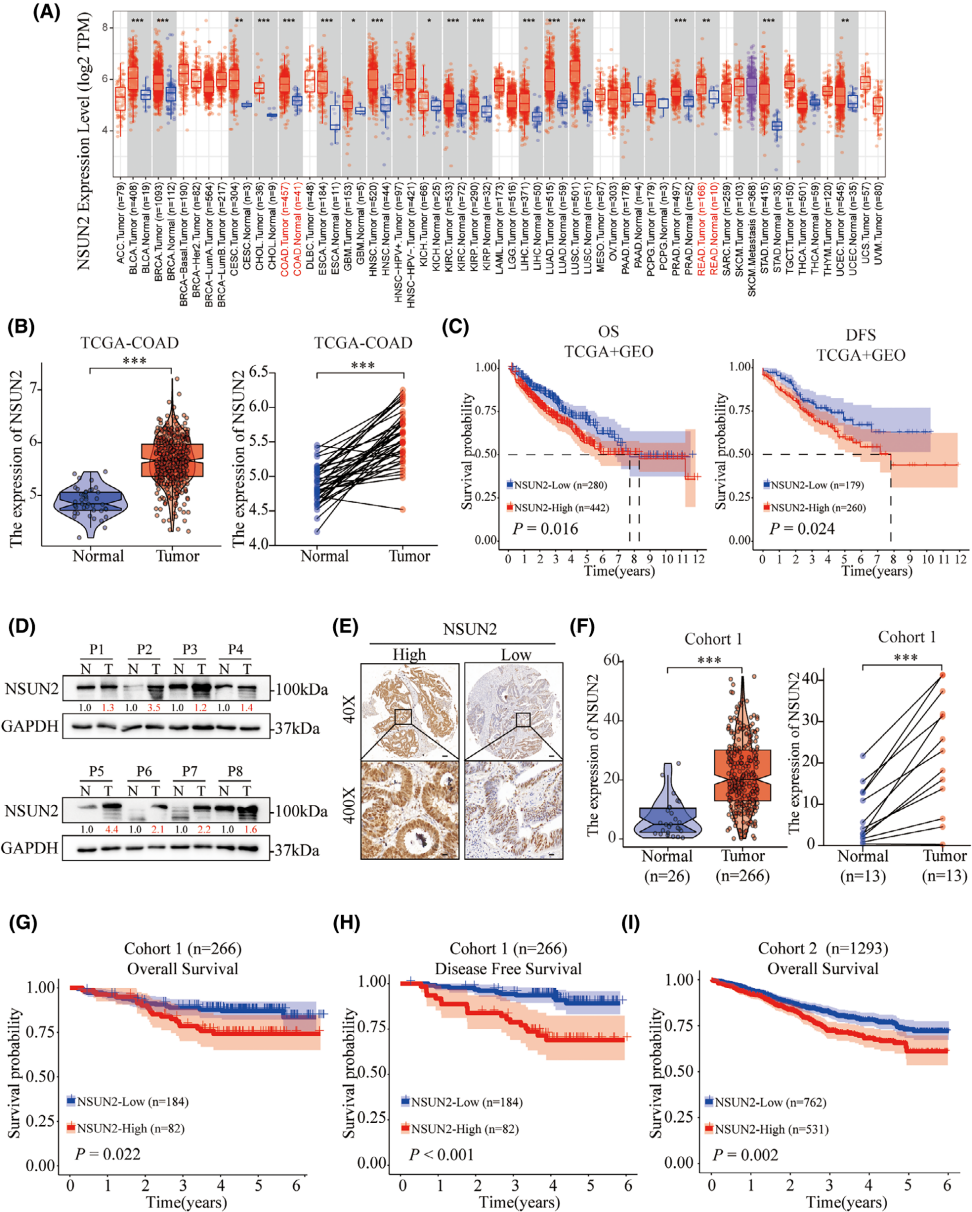
Clinical significance of NSUN2 expression in colorectal cancer (CRC). (A) The NSUN2 expression profile across 33 tumour samples and paired normal tissues. (B) NSUN2 expression was higher in CRC tumour tissues than in adjacent normal tissues. (C) Kaplan–Meier analysis of overall survival (OS) (*n* = 722, *p* = .016) and disease‐free survival (DFS) (*n* = 439, *p* = .024) in CRC patients from TCGA‐COAD and GEO (GSE17536, GSE17537, and GSE29621) databases. (D) Western blotting showed that NSUN2 protein was highly expressed in CRC tumour tissues. (E) Representative immunohistochemistry (IHC) images of NSUN2 staining in a CRC tissue microarray, bars in 40x and 400x. (F) Differential expression of NSUN2 between CRC tumour tissues and adjacent normal tissues in Cohort 1. (G,H) Kaplan–Meier analysis of OS (G) and DFS (H) according to NSUN2 expression in CRC Cohort 1. (I) Kaplan–Meier analysis of overall survival according to NSUN2 expression in CRC Cohort 2. Scale bar, 100 µm; *p < .05; **p < .01 and ***p < .001.

To evaluate the expression of NSUN2 in human tumour tissues, we noted that NSUN2 was highly expressed in the majority of solid tumours, including colon and rectal adenocarcinoma through TCGA pan‐cancer analysis (Figure 1A,B). Next, Kaplan–Meier analysis of CRC patients from TCGA‐COAD and GEO (GSE17536, GSE17537 and GSE29621) was employed to assess the role of NSUN2 in the prognosis of CRC patients. As shown in Figure 1C, high NSUN2 expression was correlated with an unfavourable prognosis in both overall survival (OS; *p* = .016) and disease‐free survival (DFS; *p* = .024). Freshly CRC tissues were then collected and detected to reveal a notably increased expression of NSUN2 protein in CRC tumours compared with normal tissues (Figure 1D). To further investigate the patterns of NSUN2 expression and its correlation with clinical pathological characteristics in patients with CRC, we collected 266 CRC paraffin tissues from First Affiliated Hospital of Wenzhou Medical University (Cohort 1) and another 1293 CRC paraffin tissues from the Second Affiliated Hospital of Wenzhou Medical University (Cohort 2) to construct CRC tissue microarrays. IHC staining for NSUN2 in the CRC tissue microarrays revealed that NSUN2 protein levels were higher in CRC tumour tissues than in adjacent normal tissues (Figure 1E,F). We then divided the patients with CRC into two groups based on the NSUN2 IHC staining scores, that is, the NSUN2‐high and NSUN2‐low groups. Clinicopathological features analysis showed that NSUN2 high expression was closely associated with positive rate of Ki‐67 (Table 1; *p* < .001). Kaplan–Meier survival analysis revealed that CRC patients in the NSUN2‐high expression group had shorter OS (*p *= .022 in Cohort 1; *p* < .002 in Cohort 2). In addition, Kaplan–Meier analysis of DFS indicated that high NSUN2 expression was associated with recurrence (Figure 1G–I). Multivariate Cox regression analysis showed that elevated NSUN2 expression served as an independent prognostic indicator for OS (Table 2; Hazard Ratio (HR), 1.72; 95% CI, 1.35–2.19; *p* < .001). Collectively, these findings suggest that NSUN2 is highly expressed in CRC tissues and correlated with an unfavourable prognosis in CRC patients.

**TABLE 1:** Clinicopathological features of NSUN2 expression in colorectal cancer (CRC) (Cohort 2).

Variables	All patients (*n* = 1293)	Low (*n* = 762)	High (*n* = 531)	*p* value
Gender				.97
Female	489	289 (37.9%)	200 (37.7%)	
Male	804	473 (62.1%)	331 (62.3%)	
Age				.574
<65	478	287 (37.7%)	191 (36.0%)	
≥65	815	475 (62.3%)	340 (64.0%)	
Tumour size				.205
<4.0 cm	561	319 (41.9%)	242 (45.6%)	
≥4.0 cm	732	443 (58.1%)	289 (54.4%)	
TNM stage				.113
0	16	11 (1.4%)	5 (.9%)	
I	245	133 (17.5%)	112 (21.1%)	
II	431	242 (31.8%)	189 (35.6%)	
III	585	366 (48.0%)	219 (41.2%)	
IV	16	10 (1.3%)	6 (1.1%)	
Differentiation				.544
Poor	66	43 (5.6%)	23 (4.3%)	
Moderate	1115	655 (86.0%)	460 (86.6%)	
Well	112	64 (8.4%)	48 (9.0%)	
Ki‐67 positive				**<. 001** ^*^
0%	1	1 (.1%)	0 (0%)	
1%–25%	25	21 (2.8%)	4 (.8%)	
26%–50%	26	18 (2.4%)	8 (1.5%)	
51%–75%	327	228 (29.9%)	99 (18.6%)	
76%–100%	914	494 (64.8%)	420 (79.1%)	

Abbreviation: TNM stage, Tumor Node Metastasis Classification.

* Statistically significant (*p* < .05).

**TABLE 2:** Univariate and multivariate Cox regression analysis of overall survival in colorectal cancer (CRC) patients (Cohort 2).

	Univariate Cox analysis		Multivariate Cox analysis	
Variables	HR (95% CI)	*p* value	HR (95% CI)	*p* value
Gender (male vs. female)	.81 (.64–1.03)	.070		
Age (≥65 vs. < 66)	2.23 (1.67–2.97)	<.001^*^	2.32 (1.74–3.10)	<.001^*^
TNM stage (III + IV vs. 0 + I + II)	2.59 (1.93–3.24)	<.001^*^	2.58 (1.99–3.34)	<.001^*^
Differentiation (poor vs. moderate + well)	2.62 (1.75–3.93)	<.001^*^	2.38 (1.58–3.58)	<.001^*^
Tumour size (≥4.0 cm vs. < 4.0 cm)	1.74 (1.34–2.27)	<.001^*^	1.50 (1.15–1.95)	.003^*^
N SUN2 expression (high vs. low)	1.48 (1.15–1.89)	.002^*^	1.66 (1.29–2.12)	.001^*^

Abbreviation: TNM stage, Tumor Node Metastasis Classification.

* Statistically significant (*p* < .05).

### 3.2 Overexpression of NSUN2 strengthens CRC cell proliferation and metastasis

**FIGURE 2:**
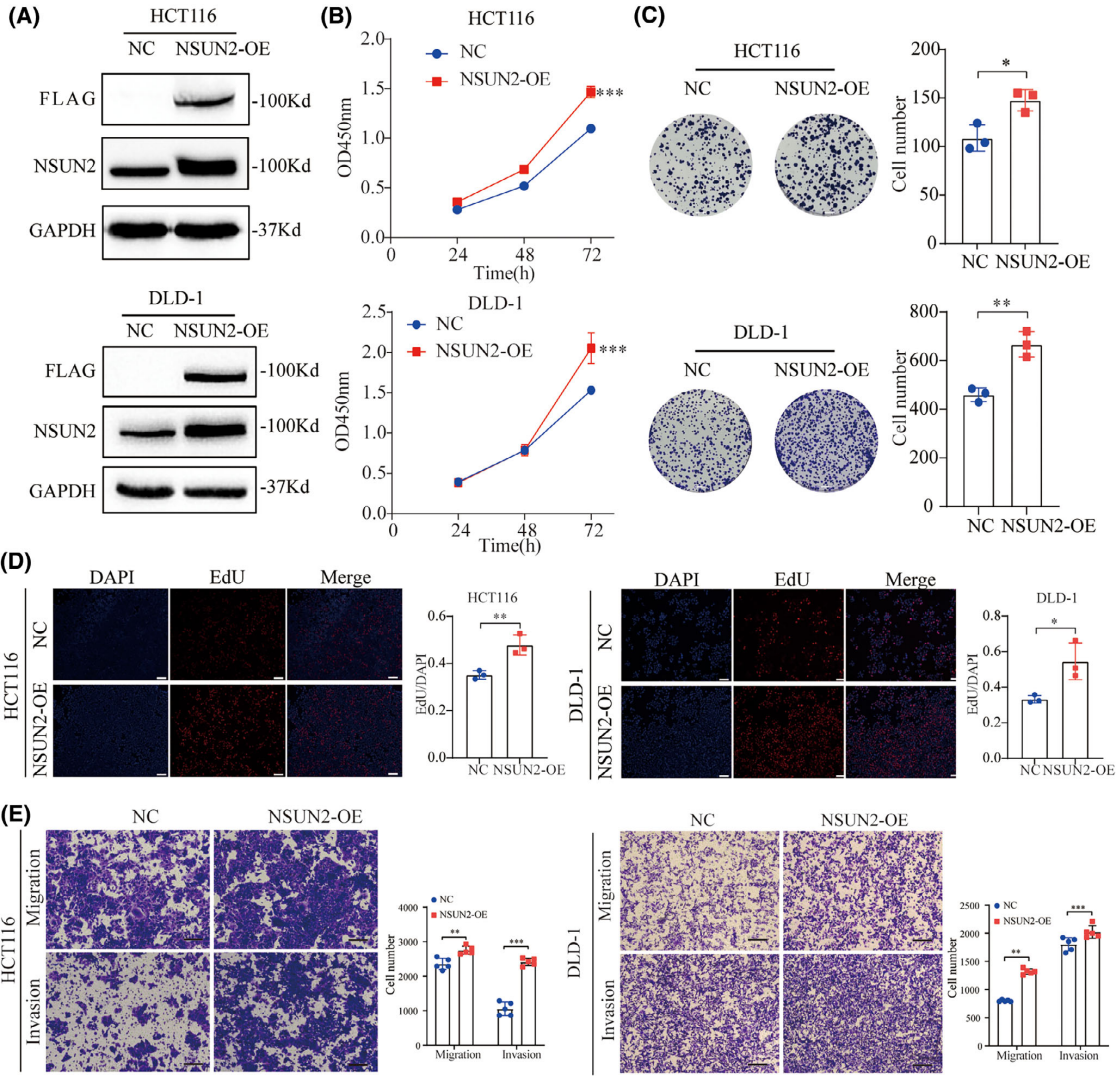
NSUN2 overexpression promotes proliferation and metastasis of colorectal cancer (CRC) cells in vitro. (A) Western blot analysis of NSUN2 overexpression efficiency in HCT116 and DLD‐1 cells. (B–D) The proliferation ability of CRC cells overexpressing NSUN2 as determined using CCK‐8, colony formation (scale bar, 3.5 mm) and EdU assays (scale bar, 100 µm). (E) The migration and invasion abilities of CRC cells overexpressing NSUN2 as determined using Transwell assays, scale bar, 100 µm. *p < .05, **p < .01 and ***p < .001.

To investigate the tumourigenic effects of NSUN2 on CRC cells, we first evaluated the expression characteristics of NSUN2 in CRC cell lines and normal cell lines (FHC). As shown in Figure S1A, NSUN2 was mainly expressed in nucleus and partially expressed in the cytoplasm. Western blot detection revealed that NSUN2 expression was relatively higher in DLD‐1 cell lines and slightly lower in HCT116 cell lines (Figure S1B). Consequently, we selected these two cell lines for subsequent experiments, and constructed stable NSUN2‐overexpressing HCT‐116 and DLD‐1 cell lines (Figure 2A). Overexpression efficiency was evaluated by western blotting (Figure 2A). We conducted CCK‐8 and EdU assays to evaluate the proliferative effects of NSUN2 in two CRC cell lines with NSUN2 overexpression. As shown in Figure 2B–D, NSUN2 overexpression significantly promoted the proliferation of DLD‐1 and HCT‐116 cells. Colony formation assays were performed to examine the long‐term effects of NUSN2 on CRC cell proliferation. We found that CRC cells overexpressing NSUN2 formed more colonies than the control CRC cells at 2 weeks (Figure 2E). Next, Transwell assays were carried out to evaluate the metastasis capabilities of CRC cells with and without NSUN2 overexpression. As shown in Figure 2E, the migration and invasion of CRC cells were markedly elevated in CRC cells overexpressing NSUN2 than in control CRC cells.

**FIGURE 3:**
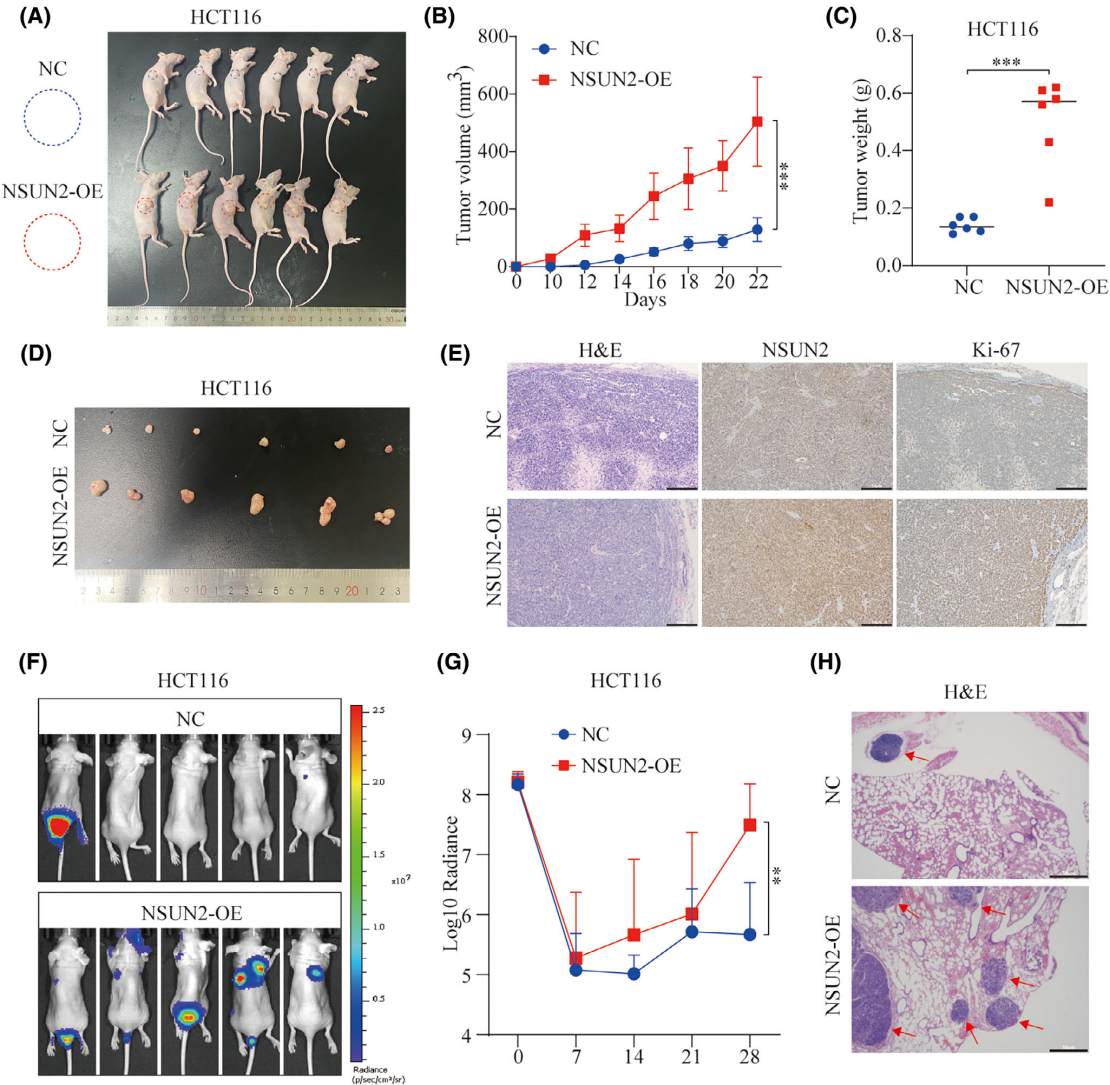
NSUN2 overexpression promotes proliferation and metastasis of colorectal cancer (CRC) cells in vivo. (A) A xenograft mouse model was constructed by subcutaneously injecting HCT116 cells into nude mice. (B,C) Growth curves and tumour weights of xenograft tumours from mice injected with HCT116‐ and HCT116 NSUN2‐overexpressing (OE) cells. (D) Representative images of xenografts. (E) Immunohistochemistry (IHC) staining of NSUN2 and Ki‐67 and haematoxylin–eosin (H&E) staining of normal control and NSUN2‐OE tumours, scale bar, 50 µm. (F,G) Lung metastasis models were constructed by injecting HCT116 cells into the tail veins of nude mice. Metastasis was verified via an IVIS system and quantified as total flux. (H) H&E staining of lung tissues in the NC and NSUN2‐OE groups, scale bar, 50 µm. *p < .05, **p < .01 and ***p < .001.

To evaluate the function of NSUN2 in CRC progression in vivo, we subcutaneously injected stable NSUN2‐overexpressing HCT‐116 cells into nude mice to establish mouse xenograft models (Figure 3A) and monitored the growth of the resulting tumours. As shown in Figure 3B–D, tumour growth in the NSUN2‐overexpression group of mice was significantly greater than that in the control group. Moreover, IHC staining confirmed that overexpression of NSUN2 significantly upregulated Ki‐67 expression in the xenograft tumour tissues (Figure 3E). The results of the xenograft assay demonstrated that overexpression of NSUN2 promoted CRC progression in vivo.

To monitor the effect of NSUN2 on metastasis of CRC cells in vivo, lung metastasis models were constructed by injecting NSUN2‐overexpressing HCT‐116 cells into the tail veins of nude mice. The NSUN2‐overexpressing HCT‐116 cells showed more obvious metastatic ability and formed more tumour foci than the control HCT‐116 cells, as shown by the photon number and haematoxylin–eosin (H&E) staining (Figure 3F,G). These results confirmed that overexpression of NSUN2 promotes metastasis of CRC cells in vivo.

### 3.3 Knockout of NSUN2 inhibits CRC tumourigenesis and progression

**FIGURE 4:**
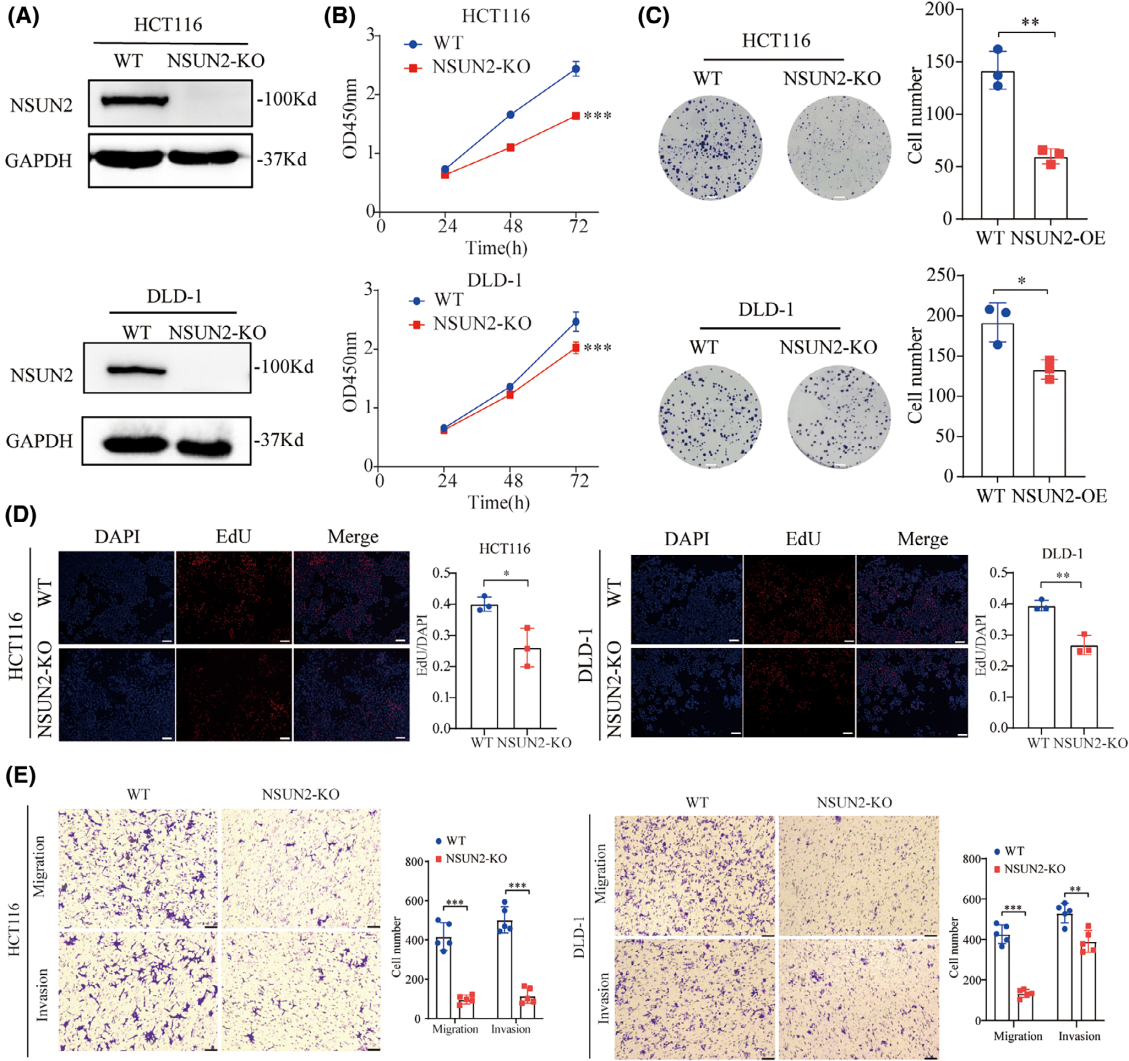
NSUN2 knockout inhibits proliferation and metastasis of colorectal cancer (CRC) cells in vitro. (A) Western blot analysis to assess NSUN2 knockout efficiency in HCT116 and DLD‐1 cells. (B–D). Proliferation of NSUN2‐knockout CRC cells was determined using CCK‐8, colony formation (scale bar, 3.5 mm) and EdU assays (scale bar, 100 µm). (E) Transwell assays to assess migration and invasion of NSUN2‐knockout CRC cells, Scale bar, 100 µm. *p < .05, **p < .01 and ***p < .001.

To further confirm the biological function of NSUN2 in CRC, we generated NSUN2‐knockout HCT‐116 and DLD‐1 cell lines using the CRISPR/Cas9 system. The knockout efficiency of NSUN2 was verified by western blotting (Figure 4A and Figure S1). CCK‐8, EdU and colony formation assays were performed to assess the effect of NSUN2 knockout on proliferative ability, and Transwell assays were performed to assess the effect of NSUN2 knockout on metastatic ability of CRC cells. As shown in Figure 3B–E, the proliferation and metastatic abilities of HCT‐116 and DLD‐1 cells were significantly suppressed by knockdown of NSUN2. Taken together, these results show that NSUN2 promotes the proliferation and metastasis of CRC cells.

### 3.4 NSUN2 promotes CRC progression via both m5C‐dependent and m5C‐independent mechanisms

**FIGURE 5:**
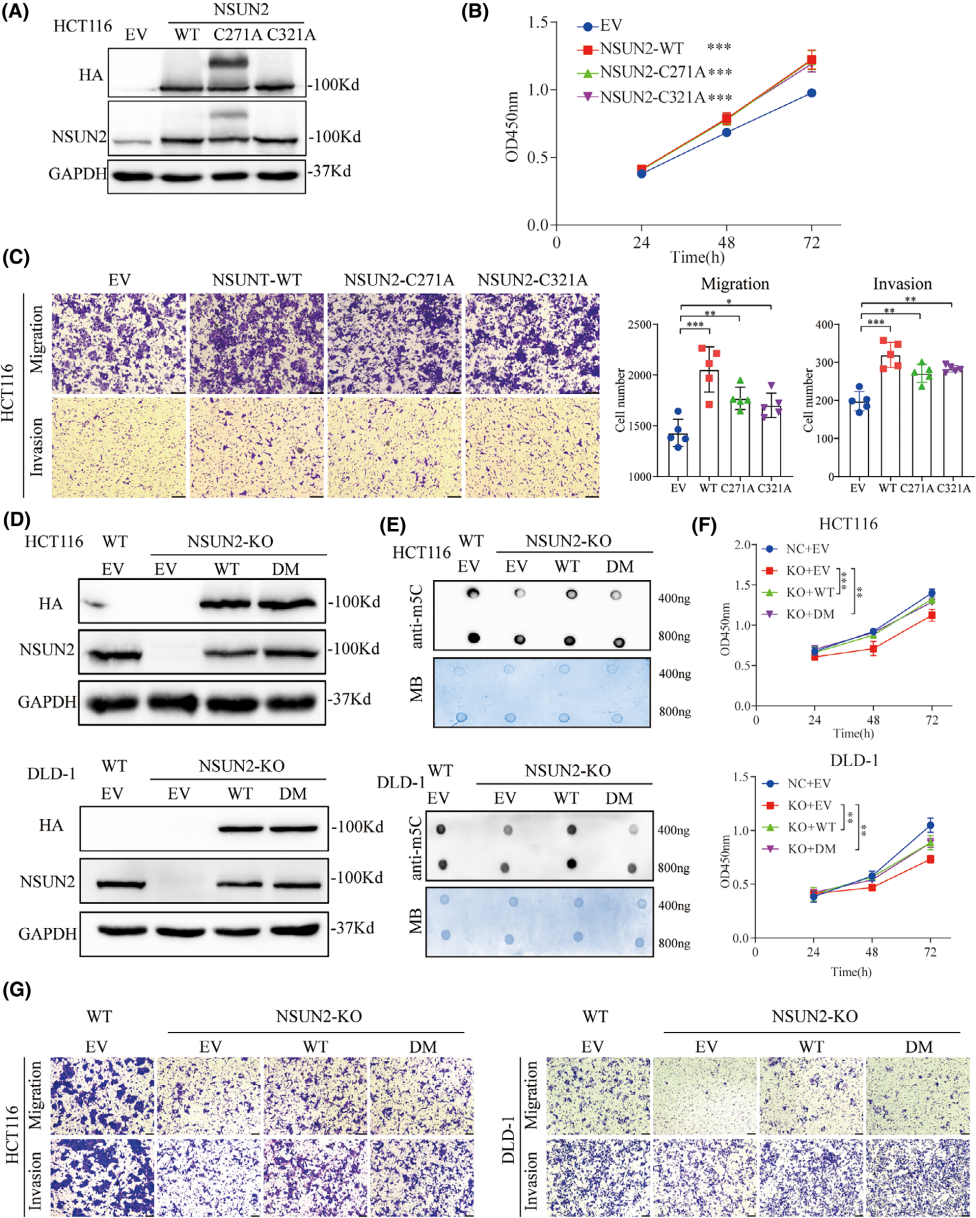
NSUN2 promotes colorectal cancer (CRC) progression via both m5C‐dependent and m5C‐independent mechanisms. (A) Western blotting to assess overexpression efficiency of wild‐type (WT) and enzymatic‐dead mutant NSUN2 in HCT116 cells. (B,C) CCK8 and Transwell assays of HCT116 cells overexpressing WT NSUN2 or the C271A and C321A enzymatic‐dead mutants. Scale bar, 100 µm. (D) Western blotting to assess overexpression efficiency of WT and enzymatic‐dead double‐mutant (DM) NSUN2 in NSUN2‐knockout HCT116 and DLD‐1 cells. (E) m5C RNA dot blot assays of NSUN2‐knockout HCT1116 and DLD‐1 cells and rescue with NSUN2 overexpression. (F,G) Proliferation and metastasis of CRC cells overexpressing NSUN2 rescued was determined via CCK‐8 and Transwell assays, scale bar, 100 µm. *p < .05, **p < .01 and ***p < .001.

NSUN2 is a classic nucleolar 5‐methylcytosine RNA methyltransferase, and its biological and oncogenic functions are generally believed to depend on its m5C catalytic activity, that is, generation of m5C at target RNAs. Previous studies have shown that both the release site (cysteine 271) and catalytic site (cysteine 321) of NSUN2 are key for its m5C methyltransferase activity. Therefore, we generated two enzymatic‐dead NSUN2 mutants by introducing point mutations at cysteine 271 and 321 (Figure 5A). Strikingly, similar to our previous study on gastric cancer cells, we found that overexpression of both the wild‐type and enzymatic‐dead mutant NSUN2 in HCT116 cells enhanced their proliferation and metastatic abilities (Figure 5B,C).

Next, we constructed an NSUN2‐DM to further confirm that the biological function of NSUN2 was not dependent on its m5C modification ability (Figure 5D). M5C dot blot assays were performed to investigate whether the level of m5C mRNA in CRC was affected by the expression of NSUN2‐WT or NSUN2‐DM. As shown in Figure 5E, m5C levels were significantly decreased when NSUN2 was knocked out, whereas m5C levels were restored when NSUN2‐WT, but not NSUN2‐DM, was overexpressed in both HCT116 and DLD‐1 cells. However, CCK‐8 and Transwell assays revealed that overexpression of both NSUN2‐WT and NSUN2‐DM rescued the proliferation and metastatic abilities of CRC cells after NSUN2 knockout (Figure 5F,G and Figure S2). Taken together, these data suggested that the non‐m5C‐dependent function of NSUN2 also plays an important role in the malignant progression of CRC.

### 3.5 The m5C‐independent function of NSUN2 activates the ErbB‐STAT3 signalling pathway through its interaction with CUL4B

**FIGURE 6:**
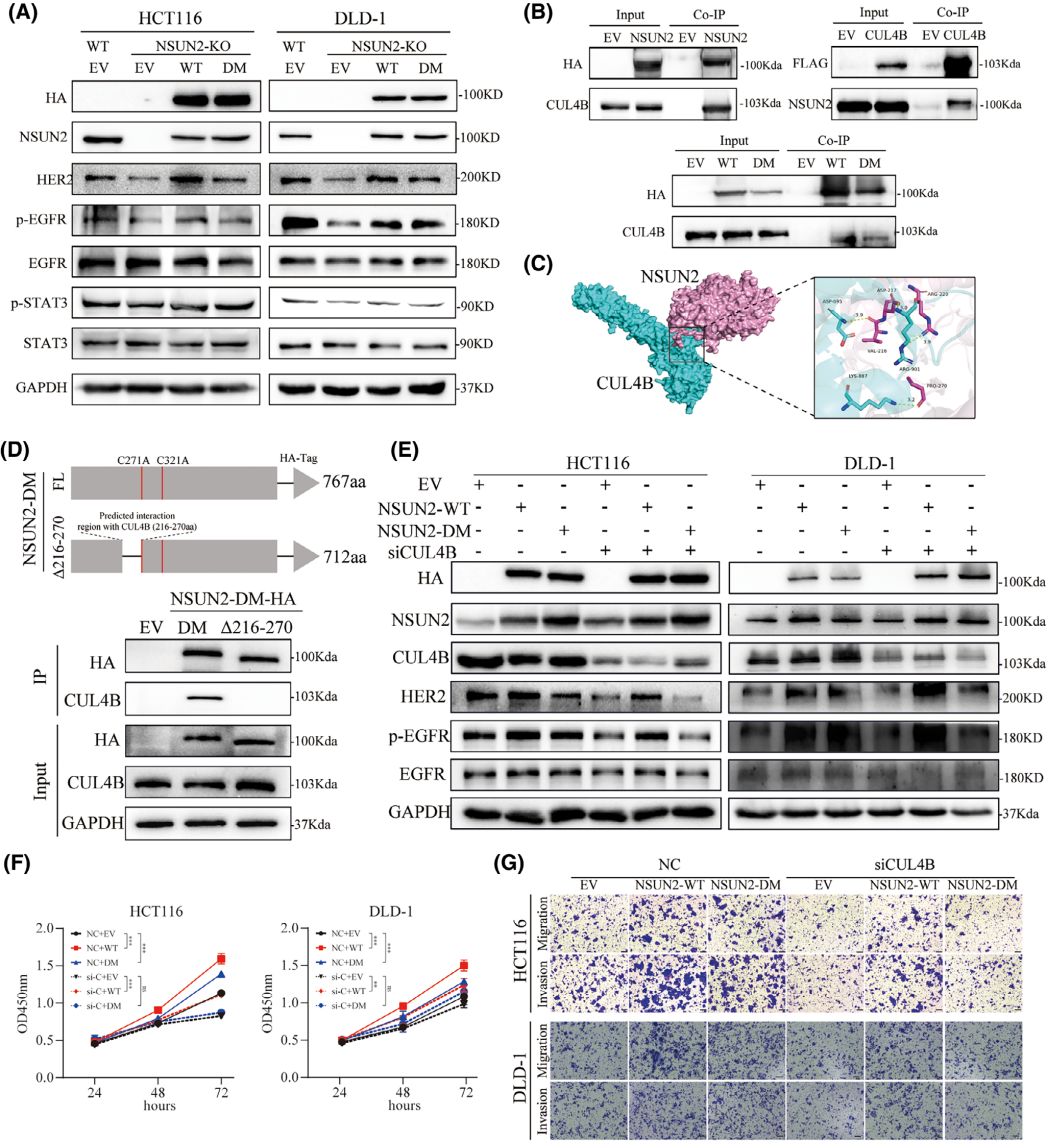
The m5C‐independent function of NSUN2 activates the ErbB‐STAT3 signalling pathway through its interaction with CUL4B. (A) Western blot analysis of ErbB signalling pathway activation in NSUN2‐knockout HCT116 and DLD‐1 cells with and without NSUN2 overexpression. (B) Co‐IP experiments showing the interaction between (wild‐type [WT] and double‐mutant [DM]) NSUN2 and CUL4B. (C) Molecular docking pattern diagram of NSUN2 and CUL4B. (D) Schematic representation of NSUN2Δ216‐270 deletion mutant plasmid construction and validation of its interaction with CUL4B via Co‐IP. (E) Western blot analysis of the changes in the ErbB signalling pathway changes in CUL4B‐knockdown cells and these cells following NSUN2 (WT and DM) rescue. (F,G). Proliferation and metastasis of CUL4B‐knockdown CRC cells and following NSUN2 (WT and DM) rescue. Scale bar, 100 µm. *p < .05, **p < .01 and ***p < .001.

To investigate the molecular mechanism underlying the m5C‐independent promotion of CRC progression for NSUN2, we attempted to identify the signalling pathways downstream of NSUN2. Previous research have found that activation of the ErbB pathway is closely correlated to m5C levels and the expression levels of its regulators. Therefore, we explored whether an enzymatic‐dead NSUN2 mutant could upregulate the ErbB signalling pathway. We noted substantial decreases in the expression levels of HER2, phosphorylated EGFR and phosphorylated STAT3 in NSUN2‐knockout CRC cells, whereas NSUN2‐WT overexpression reversed the reductions in these levels. As anticipated, complementation with m5C enzymatic‐dead NSUN2‐DM also restored these levels, although the effect was not as pronounced as that for NSUN2‐WT (Figure 6A). These results indicate that NSUN2 activates the ErbB signalling pathway in both an m5C‐dependent and m5C‐independent manner.

In pursuit of understanding the mechanism underlying the m5C‐independent regulation of the ErbB signalling pathway by NSUN2, we attempted to identify the proteins that interact with NSUN2. We found that CUL4B bound tightly to NSUN2 using co‐IP (Figure 6B). Additionally, overexpression of CUL4B in CRC cells also immunoprecipitated NSUN2 (Figure 6B), and both NSUN2‐WT and NSUN2‐DM interacted with CUL4B. We used a molecular docking model to predict the binding affinity and interacting regions between NSUN2 and CUL4B (Figure 6C). The computational analysis indicated a stability of −239.9 kcal/mol for these two proteins. Subsequently, to validate the domain that interacts with CUL4B, we constructed a mutant NSUN2 with a 216–270 amino acid deletion. As shown in Figure 6D, deletion of the 216–270 amino acid segment resulted in the loss of interaction between NSUN2 and CUL4B, thereby confirming that this region of the NSUN2 protein is essential for its interaction with CUL4B. These results indicate that NSUN2 interacts with CUL4B independent of its m5C modification activity and that it may exert its biological functions through CUL4B.

A preceding investigation has delineated that HER2 is a downstream target of CUL4B in gastric cancer. Therefore, we speculated that NSUN2 might interact with CUL4B to regulate the expression of the HER2 signalling pathway. CUL4B, a constituent of the largest family of ubiquitin E3 ligases, is implicated in a myriad of biological processes. Previous studies have shown that CUL4B is primarily involved in the formation of the CRL4B complex, which recognizes and ubiquitinates downstream substrate proteins. Recent studies have shown that CUL4B directly modulates downstream genes expression at the transcriptional level. Therefore, we evaluated the biological effects exerted by CUL4B in CRC cells (Figure S3). CUL4B knockdown via siRNA markedly suppressed both the proliferation and metastatic capabilities of CRC cells. Next, we knocked down CUL4B in CRC cells while simultaneously overexpressing either NSUN2‐WT or NSUN2‐DM and evaluated the changes in the ErbB signalling pathway and malignant phenotype of CRC cells. The expression levels of HER2, phosphorylated EGFR and phosphorylated STAT3 decreased upon CUL4B knockdown, and only overexpression of NSUN2‐WT restored the levels of these proteins. Overexpression of NSUN2‐DM did not rescue the defects in the ErbB signalling pathway (Figure 6D). Similarly, overexpression of NSUN2‐WT partially restored the metastatic ability of CUL4B‐knockdown HCT116 and DLD‐1 cells, whereas overexpression of NSUN2‐DM did not (Figure 6E–G and Figure S3C). Collectively, these findings indicated that the m5C‐independent functions of NSUN2 might depend on CUL4B expression.

### 3.6 NSUN2 enhances the therapeutic efficacy of lapatinib in CRC

**FIGURE 7:**
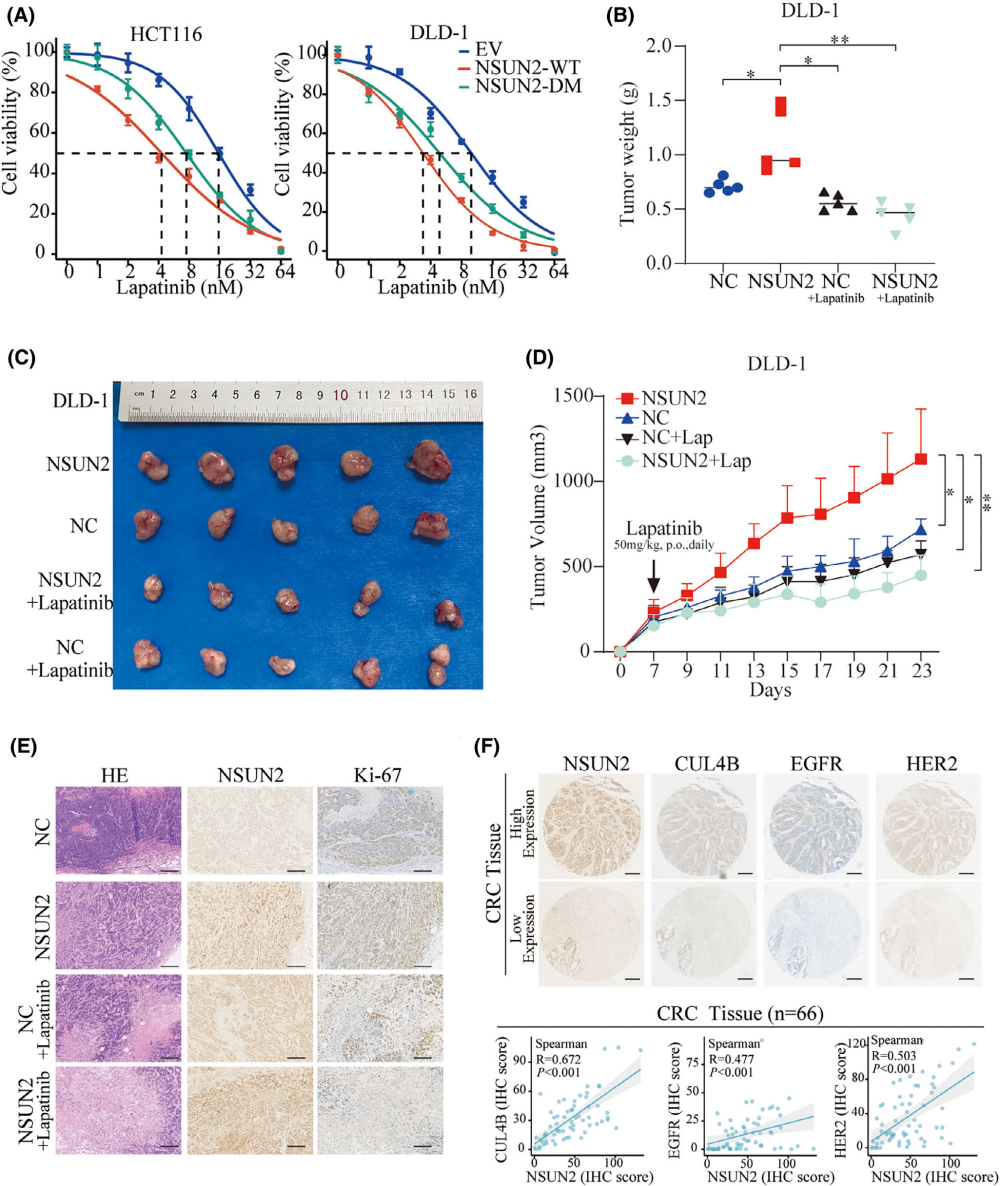
NSUN2 enhances the sensitivity of colorectal cancer (CRC) to lapatinib. (A) IC50 analysis of lapatinib in NSUN2‐WT‐ and NSUN2‐DM‐overexpressing CRC cells. (B–D) Xenograft tumour model mice were established by injection with DLD‐1 cells overexpressing NSUN2. The mice were treated with lapatinib (50 mg/kg daily) via oral gavage. Tumour weight (B), representative tumour images (C) and growth curves (D) are shown. (E) Immunohistochemistry (IHC) staining for NSUN2 and Ki‐67 and haematoxylin–eosin (H&E) staining of xenograft tumour tissues, scale bar, 200 µm. (F) Representative IHC staining images and co‐expression analysis of NSUN2, CUL4B, EGFR and HER2 in CRC tissues (*n* = 66), scale bar, 250 µm.*p < .05, **p < .01 and ***p < .001.

The application of molecular targeted inhibitors has brought a revolution in cancer therapy. These agents exhibit higher selectivity and fewer side effects as they specifically target molecular markers on cancer cells, thereby inhibiting tumour growth and metastasis. Therefore, we aimed to identify inhibitors that could target CRC with high NSUN2 expression. Among these, lapatinib, a potent dual EGFR‐HER2 inhibitor, attracted our attention. We found that overexpression of NSUN2‐WT increased the sensitivity of CRC cells to lapatinib. Interestingly, a similar effect was observed in cells overexpressing the m5C enzymatic‐dead NSUN2‐DM (Figure 7A). To further validate the therapeutic effect of lapatinib on CRC with high NSUN2 expression, a xenograft model was established in nude mice through the subcutaneous implantation of NSUN2 overexpression DLD1 cells. We then treated the mice with lapatinib, (orally, at 50 mg/kg per day). Evaluation of subcutaneous tumour volume and their growth curves showed that although the differences between the NC‐ and NSUN2‐overexpressing groups were not significant after lapatinib treatment, the size of NSUN2‐overexpressing tumours was significantly reduced compared with the untreated NSUN2 group after lapatinib treatment (Figure 7B–D). H&E staining and assessment of Ki‐67 levels using IHC staining showed the level of Ki‐67 in the subcutaneous tumour tissue in each group (Figure 7E). Additionally, the expression correlations of NSUN2, CUL4B, EGFR and HER2 were examined via IHC in xenografts and CRC patient tissues. The results indicated a positive correlation in the expression levels of these proteins (Figure S4 and Figure 7F). Spearman's analysis revealed significant positive correlations between NSUN2 and CUL4B (*R* = .672; *p* < .001), EGFR (*R* = .477; *p* < .001) and HER2 (*R* = .503; *p* < .001) in CRC patients (Figure 7E). Collectively, these findings revealed that NSUN2 upregulates EGFR/HER2‐STAT3 pathway and enhances the therapeutic efficacy of lapatinib in CRC.

## 4 DISCUSSION

**FIGURE 8:**
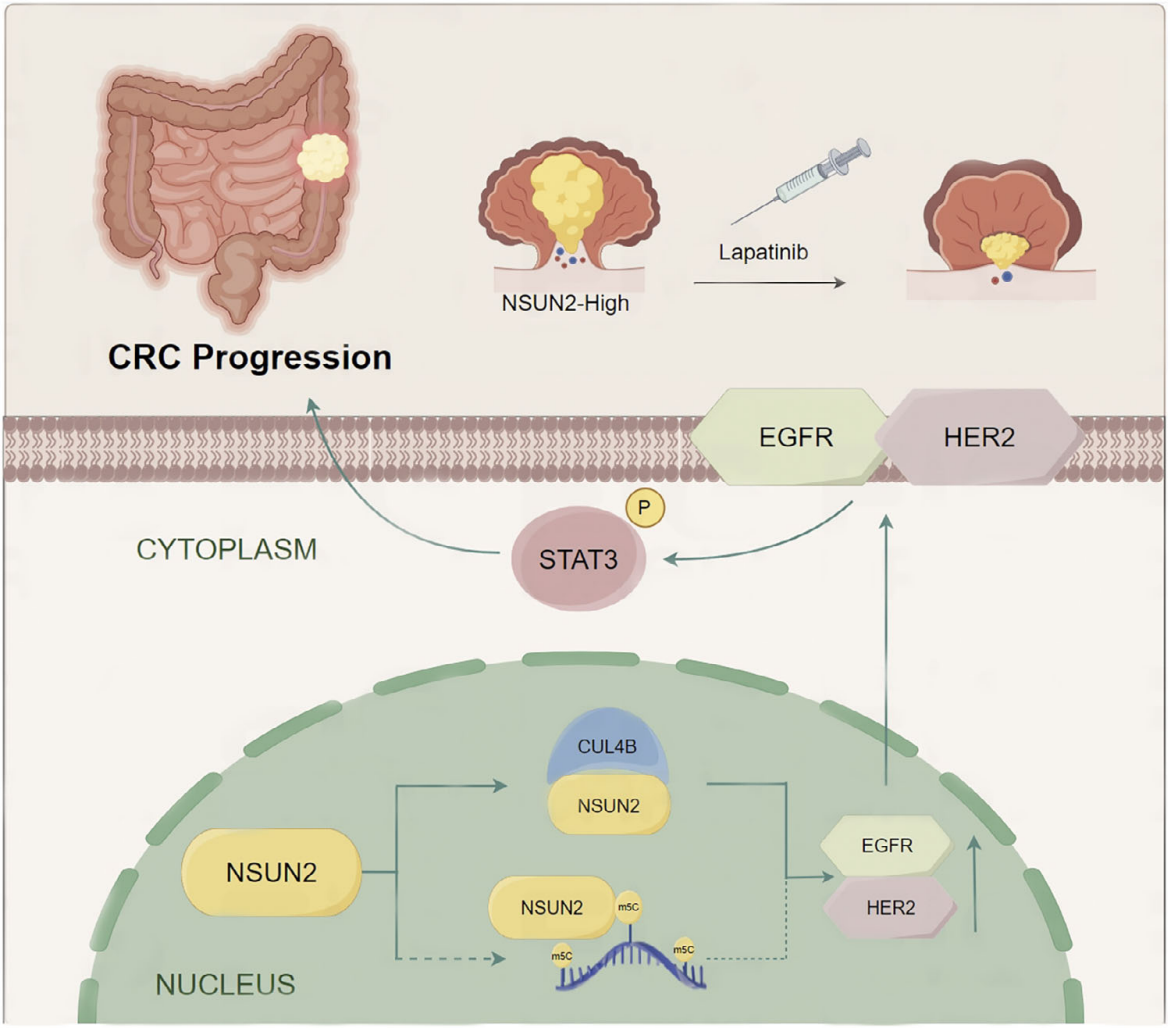
Schematic illustration summarizing the mechanism by which NSUN2 promotes colorectal cancer (CRC). NSUN2 is highly expressed in CRC and promotes malignancy by interacting with CUL4B to activate the ErbB‐STAT3 signalling pathway in an m5C‐independent manner. NSUN2‐mediated elevation of EGFR/HER2 expression enhances the sensitivity of CRC to lapatinib.

CRC is recognized as one of the most frequent primary cancers globally, and it frequently progresses rapidly.^20^ Over recent decades, treatments for CRC, including surgery, chemotherapy, and targeted therapies, have rapidly improved. However, the clinical outcomes for patients with advanced CRC have not significantly improved. Therefore, molecular biomarkers and new therapeutic targets are urgently needed to develop effective targeted therapies. Here, our findings demonstrated that the RNA m5C methyltransferase NSUN2 is overexpressed in CRC and correlates with an unfavourable prognosis. In addition, we identified a novel mechanism underlying the biological functions of NSUN2 in CRC. Independent of its m5C methyltransferase activity, NSUN2 interacts with CUL4B to co‐regulate the EGFR/HER2 pathway, thereby mediating the sensitivity of CRC to lapatinib (Figure 8).

Accumulating evidence has revealed that post‐transcriptional RNA modifications are crucial for a variety of biological processes, and dysregulation of epigenetic modifications is closely associated with various diseases, including cancers. As key orchestrators of RNA modifications, changes in the expression level or catalytic activity of methyltransferases (writers), binding proteins (readers) and demethylases (erasers) can directly lead to dysregulation of the RNA modification landscape.^21^ NSUN2, the methyltransferase that catalyzes m5C modification, has been reported to exert its biological functions primarily in an m5C‐dependent manner, such as epidermal stem cell self‐renewal, tumour cell stemness and tumourigenesis.^22^, ^23^, ^24^ For example, Blanco et al.^25^ discovered that NSUN2‐mediated post‐transcriptional RNA methylation is a key process associated with regulation of the regeneration and differentiation of epidermal stem cells. Chen et al.^26^ demonstrated that NSUN2 functions as a glucose sensor that maintains tumour cell stemness via its m5C activity. Furthermore Su et al.^[^ ^27^ ^]^ found that NSUN2 stabilizes GRB2 mRNA through m5C modification to facilitate the progression of oesophageal cancer. Zhu et al.^28^ showed that androgen receptor (AR) transcriptionally regulates expression of NSUN2 in prostate cancer and that NSUN2 mediates the stabilization of AR mRNA via m5C methylation, thereby initiating a positive feedback mechanism to foster tumour progression. Chen et al.^29^ demonstrated that NSUN2 inhibits ferroptosis and promotes endometrial cancer malignancy by regulating the level of m5C SLC7A11 mRNA. Chen et al.^30^ also showed that NSUN2 reprogrammed the metabolism of CRC via a NSUN2/YBX1/m5C‐ENO1 positive feedback loop.^30^ Another study showed that NSUN2 promotes CRC progression by enhancing stabilization of SKIL mRNA through m5C modification. In their study, an enzymatic‐dead mutant of NSUN2 had no effect on CRC tumourigenesis.^31^ In the present study, we confirmed that NSUN2 exhibits increased expression in CRC and is closely associated with poor outcomes in two distinct cohorts. Furthermore, NSUN2 enhanced the proliferation and metastatic abilities of CRC cells both in vivo and in vitro. These results indicate that NSUN2 may act as an oncogene in CRC and provide new insights into the pathogenesis of CRC progression.

From a biological phenotypic perspective, our findings are largely consistent with previous results, in that NSUN2 was highly expressed in CRC and played a vital role in enhancing its malignant properties. However, it is interesting to note that our research uncovered a novel finding that the tumourigenic effects of NSUN2 in CRC were not entirely reliant on its m5C enzymatic activity, which contrasts others' reports. We initially observed this phenomenon in gastric cancer, where m5C enzymatic‐dead mutants of NSUN2 (C271A and C321A) partially promoted the proliferation and metastasis of gastric cancer cells.^9^ In this study, we constructed an m5C enzymatic‐dead NSUN2‐DM and confirmed that expression of NSUN2‐DM did not restore the decrease in m5C levels caused by NSUN2 knockout. Nonetheless, it partially rescued the malignant phenotype of CRC cells, indicating that NSUN2 exerts its effects through m5C‐independent mechanisms. Zou et al. have similarly investigated the effects of NSUN2‐DM on CRC cell proliferation and found that there was a certain upward trend in the DM group compared to the control group, although the observed differences were not statistically significant.^31^ In this study, the authors used an shRNA model to knock down the expression of NSUN2 in CRC cells, whereas in our study, we assessed the biological roles of NSUN2‐DM using a NSUN2 knock‐out model, which may account for the discrepant findings of the two studies. We speculate that these differences could be related to the basal levels of NSUN2‐WT in CRC cells. Subsequent to NSUN2 knockdown using shRNA, a certain of residual amount NSUN2 may contribute to maintain its non‐m5C‐dependent biological activities, thereby explaining our failure to detect any significant biological activity upon NSUN2‐DM complementation. Contrastingly, in NSUN2‐KO cells, complementation with NSUN2‐DM was found to promote more pronounced biological activity. In addition, given that previous research has highlighted the importance of the m5C activity of NSUN2 in the progression of CRC, there has been comparatively little research assessing the biological functions of NSUN2‐DM. Given that gastrointestinal tumours, particularly CRC, are directly exposed to a diverse range dynamic stimulatory factors, including nutrients, microorganisms, and drugs, this accordingly highlights the potentially complex nature of the regulatory mechanisms of NSUN2 in the progression of CRC.^19^ In addition, multiple strands of evidence have indicated that NSUN2 may have m5C‐independent biological functions with respect to cell cycle progression. An early study found that in interphase, NSUN2 functions as an RNA methyltransferase to ensure translation efficiency, whereas in cell division, it forms an RNA–protein complex with 18S rRNA that regulates spindle assembly during mitosis, thereby promoting proliferation, and its function during cell division is independent of its methyltransferase activity.^16^ As for viral infection regulation, NSUN2 has been reported not only to facilitate enterovirus 71 replication via m5C modification but also to interact with the viral‐encoded VP1 protein and maintain its stabilization in a m5C‐independent manner.^32^ Recent research has confirmed that the METTL3/METTL14 methyltransferases act as a crucial intermediary for CRL4 complex, facilitating the polyubiquitination and subsequent proteasomal degradation of SUV39H1/H2.^17^ Another study on m6A found that METTL16 promotes translation initiation through direct binds to eIF3a/b and rRNA in an m6A‐independent manner.^18^ These research results support our findings and indicate that the methylation‐independent biological functions of not only NSUN2 but also other RNA methyltransferases warrant further exploration.

The ERBB signalling pathway is one of the most frequently activated and upregulated pathways in oncogenesis, and it plays crucial roles in diverse biological and pathogenic processes, including cell proliferation, metastasis and chemosensitivity. The ERBB family of receptor tyrosine kinases comprise four receptors: EGFR, HER2, HER3 and HER4. Two of these, EGFR and HER2, which regulate various tumourigenic processes through multiple signalling pathways, including the PI3K/Akt, STAT3 and Ras/Raf/MEK/ERK pathways, have garnered widespread attention in CRC.^33^, ^34^ Although overexpression or activation of EGFR and HER2 is associated with poor prognosis in patients with CRC, the underlying mechanisms are not well understood. Another research found that m5C regulators are closely associated with the ErbB‐PI3K‐Akt signalling pathway in gastrointestinal cancer.^35^ In addition, ALYREF‐mediated m5C modification of EGFR mRNA stabilized EGFR mRNA and activated the STAT3 signalling pathway. Another study had revealed that silencing NSUN2 in lung cancer led to significant downregulation of EGFR expression.^10^ In our study, we found that overexpression of NSUN2 significantly activated the EGFR/HER2‐STAT3 signalling pathway in both m5C‐dependent and m5C‐independent manners. Recent research identified CUL4B as an upstream regulator of HER2 that upregulates HER2 expression by repressing miR‐125a transcription.^36^ CUL4B is upregulated in diverse solid tumours, such as gastric cancer, CRC and pancreatic cancer. CUL4B exerts its biological functions by mediating substrate degradation via the ubiquitination pathway. In addition, CUL4B can also form CRL complexes with DDB1, ROC1 and DCAF and thereby directly participate in the transcriptional regulation of downstream genes.^37^, ^38^, ^39^ Here, we demonstrated that NSUN2 interacts with CUL4B in an m5C‐independent manner and that silencing of CUL4B results in a near loss of NSUN2 m5C‐independent activity, suggesting that the m5C‐independent function of NSUN2 may be mediated by CUL4B.

Targeting HER2 expression with trastuzumab treatment partially reversed the malignant progression of gastric cancer caused by CUL4B overexpression.^36^ Therefore, we explored whether NSUN2‐induced activation of EGFR/HER2 in CRC could be targeted by ErbB‐targeted inhibitors. Lapatinib is a potent dual inhibitor of EGFR and HER2 tyrosine kinases; it inhibits the tyrosine kinase activity of these receptors, thereby preventing signal transduction, proliferation and survival of tumour cells. Lapatinib treatment also inhibits serine and tyrosine phosphorylation of STAT3.^40^ Combined with trastuzumab, lapatinib is used as a second‐line treatment for advanced HER2‐positive breast cancer patients.^41^, ^42^ Trastuzumab plus lapatinib has been evaluated in a phase 2 clinical trial for the treatment of HER2‐positive metastatic CRC, demonstrating an objective response rate of 28%, with one complete response.^43^ In our study, we showed that NSUN2 increased expression and activation of the ErbB signalling pathway in an m5C‐dependent and m5C‐independent manner. Furthermore, CRC cells overexpressing NSUN2 displayed enhanced sensitivity to lapatinib, pointing to a potential novel therapeutic target for the prevention, control and treatment of CRC.

In summary, our study revealed that NSUN2 is significantly upregulated and associated with poor prognosis in patients with CRC. NSUN2 promotes the progression and metastasis of CRC in vitro and vivo in an m5C‐dependent and m5C‐independent manner. Mechanistically, NSUN2 interacts with CUL4B to promote activation of the EGFR/HER2‐STAT3 signalling pathway. In addition, overexpression of NSUN2 in CRC promoted sensitivity to lapatinib (Figure 7F). In conclusion, NSUN2 may be a promising diagnostic biomarker and potential therapeutic target for CRC. In addition, our findings in this study provide novel perspectives for the investigation of RNA methyltransferases, highlighting the importance of considering not only the catalytic roles of these proteins in RNA modification but also their broader biological functions. Additionally, when developing drugs targeting NSUN2 in cancer therapy, future efforts should focus not only on blocking its RNA modification pathways but should also consider its m5C‐independent functions.

Nevertheless, despite our important findings, this study does have certain limitations. Notably, we characterized the biological functions of the NSUN2 m5C enzyme‐dead mutant in only two CRC cell lines, namely, DLD1 and HCT116, thereby, highlighting the need for parallel assessments using other cell lines or animal assays. Furthermore, although we succeeded in demonstrating an association between high NSUN2 expression and sensitivity to lapatinib in CRC, this association needs to be further validated based on correlations between the clinical levels of NSUN2 expression and the therapeutic efficacy of lapatinib, cetuximab or herceptin. In addition, although we established that NSUN2 interacts with CUL4B, thereby promoting an upregulation of the ErbB/STAT3 signalling pathway in CRC, the associated mechanisms have yet to be elucidated, and thus warrant further investigation. Nevertheless, on the basis of our findings in this study, we have provided substantial evidence to indicate the m5C‐independent function of NSUN2 in the progression of CRC, and these limitations should not unduly detract from the significance of these findings.

## AUTHOR CONTRIBUTIONS

*Study concept and design*: Zhiguang Zhao, Xiangyang Xue, Xiaodong Chen and Xian Shen. *Experiment*: Yuanbo Hu, Chenbin Chen, Kezhi Lin, Xinya Tong, Tingting Huang, Tianle Qiu and Jun Xu. *Analyze bioinformatics data*: Xietao Chen, Wangkai Xie, Xiangwei Sun, Shiyu Feng and Chenbin Chen. *Data collection*: Kezhi Lin, Mingdong Lu and Zhiguang Zhao. *Writing and original draft preparation*: Yuanbo Hu.

## CONFLICT OF INTEREST STATEMENT

The authors declare no conflicts of interest.

## ETHICS STATEMENT

The study was approved by the Review Board of the First (KY2022‐202) and Second (2021‐K‐42‐01) Affiliated Hospital of Wenzhou Medical University and Ethics Committee on Laboratory Animals of Wenzhou University (WZU‐2024‐074/075). All patients were informed of the study and had signed informed consent

## Supporting information



Supporting Information

## Data Availability

All data generated or analyzed during this study are included in the article and are available from the corresponding author upon reasonable request.
