# Supplementary material for: NSUN2 promotes colorectal cancer progression and increases lapatinib sensitivity by enhancing CUL4B/ErbB‐STAT3 signalling in a non‐m5C manner
Source: Clin Transl Med. 2025 Mar 28;15(4):e70282. doi: 10.1002/ctm2.70282 (PMC11953055; doi:10.1002/ctm2.70282)
Supplement: Supplementary file 1 — Supporting Information [file CTM2-15-e70282-s001.docx]

**Supplementary Methods**

**Public species dataset source and bioinformatics analysis**

RNA expression data and the corresponding clinicopathological data were procured from The Cancer Genome Atlas (TCGA; <https://portal.gdc.cancer.gov/>) and Gene Expression Omnibus (GEO, <https://www.ncbi.nlm.nih>.gov/geo/; GSE17536, GSE17537 and GSE29621). In addition, 647 CRC tissues and 51 normal tissues from TCGA-COAD and TCGA-READ were included in differential expression analysis. Combining the TCGA and GEO data, a total of 722 samples were included in survival analysis after batch correction and removal of samples lacking clinical information.

**Immunohistochemistry (IHC)**

The NSUN2 expression levels in CRC tumor tissue and normal tissue were assessed using immunohistochemistry as previously described. Briefly, the sections were treated with anti-NSUN2 primary antibody, then with HRP-conjugated secondary antibody, visualization by DAB ((Dako, Carpinteria, CA, USA)) and then counterstaining by hematoxylin. Then, IHC-score (mean IOD) was calculated by the intensity of staining and the proportion of labeled tumor cells by Image Pro-Plus as previously described.

**Cell culture and transfection**

Human colorectal cancer cell lines DLD-1, HCT116 were purchased from National Collection ofAuthenticated Cell Cultures (Shanghai, China). The cells were cultured in Dulbecco’s Modified Eagle Medium (DMEM, Gibco; HCT116) and RPMI-1640 Medium (Gibco; DLD-1), supplemented with 10% fetal bovine serum (FBS; Sigma). All the cell lines were incubated in 37°C at a humidified 5% CO2.

**Cell Transfections**

The HA-tagged NSUN2 and FLAG-tagged CUL4B ORF was subcloned PCR-amplified and insert into pcDNA3.1 (+) vector. The mutants of NSUN2 (C271A, C321A, DM, and the relevant Cas9-gRNA resistant mutants) were obtained via homologous recombination. All plasmids were validated by DNA sequencing. For transient small interfering RNA (siRNA) transfection, CUL4B siRNA (si-CUL4B-1, si-CUL4B-2, and si-CUL4B-3), and a negative control (si-NC) were synthesized by RiboBio (Guangzhou, China; Table S1). Lipofectamine 2000 (Invitrogen) was used for cell transfection according to the manuals.

CRC cell lines stably overexpressing NSUN2 were generated by infecting NSUN2-FLAG lentivirus (HANBIO, Shanghai) and selected by puromycin. And the NSUN2-knockout CRC cell lines were constructed using the CRISPR-Cas9 gene-editing system, with lentivirus containing Cas9-guide RNA targeting sequences (5’-TGTTCTCCTTGACGATCTCG-3’) (HANBIO). Next, the cells were selected by puromycin and then monocolonies were picked.

**Immunofluorescence (IF)**

After transfection, cells were washed with cold PBS, fixed in 4% paraformaldehyde for 15 minutes. 0.5% Triton-X100 was used for cell membranes permeabilion. The cells were blocked with 5% goat serum for 30min at 37°C and incubated with primary anti-HA and anti-NSUN2 at 4°C overnight. The cells were washed with PBST and exposed to secondary antibodies for 1h at 37°C. Afterwards, the cells were washed with PBST and incubated with DAPI for 3 min. All images were captured using a fluorescence microscope.

**Co-immunoprecipitation**

For Co-IP assays, the transfected cells were lysed with NP-40 containing a protease inhibitor cocktail (MCE, Shanghai). The supernatant was retrieved and subjected to incubation with Anti-HA Magnetic Beads (Thermo Fisher Scientific Inc, USA) and Anti-FLAG Magnetic Beads (Beyotime) overnight at 4℃. The beads underwent three rounds of washing and incubated with diluted elution buffer at 95℃ for 5 min. The results were analyzed by SDS-PAGE.

**Western blotting**

Cells were harvested and prepared in RIPA lysis buffer (Beyotime, Shanghai) containing protease inhibitor cocktail (MCE). Whole-cell lysates were added to SDS-PAGE and transferred to PVDF membrane (Bio-Rad, Hercules, CA, USA). Following the blocking step and subsequent incubation with the specified primary and secondary antibodies, the results were observed by the Bio-Rad Imaging System. Anti-HA (1:1000, CST), anti-NSUN2 (1:5000, Proteintech, 20854‐1‐AP), anti-GAPDH (1:1000, Proteintech, 60004-1-Ig), anti-CUL4B (1:4000, Proteintech, 60004-1-Ig), were used. All the protein expression levels were normalized base on GAPDH.

**Cell proliferation and colony formation assays**

For Cell Counting Kit-8 (CCK8) assay，5000 cells per well were seeded into 96 well plates with 5 replicates and preincubated overnight. After transfection for 24h, 48h, 72h, the medium was replaced with fresh medium containing 10% CCK-8 reagent (Dojindo Molecular Technologies, Kumamoto, Japan), and then incubated for 3 hours. The absorbance was detected at 450 nm. All experiments were carried out in triplicate.

For colony formation assays, transfected cells were plated at a density of 500 cells per well in 6 well plates and cultured for 2 weeks. Subsequently, cells were fixed with 4% paraformaldehyde for 15 min and stained with 0.1% crystal violet for 15min. The number of colonies was counted under a microscope.

**5-Ethynyl-2’-deoxyuridine (EdU) assay**

For EdU assay, 8000 cells per well were added into 96 well plates. After transfection, EdU (50μM, Cell Light EdU DNA imaging Kit, RiboBio) was added to each well and incubate at 37°C for 2h. CRC cells were fixed with 4% paraformaldehyde for 30 min, permeabilized with 0.5% Triton-X100-PBS for 10 min. After staining by EdU Apollo®567 for 30 min and Hoechst 33342 for 20 min, the proportion of EdU-positive cells were visualized with fluorescence microscopy.

**Transwell migration and invasion assays**

For the transwell migration assays, 2×10^5^ transfected cells were resuspended in serum-free medium (200 μL) and plated in the apical chamber of a Transwell system (Corning, NY, USA) for migration. The cells were placed on Matrigel-coated bottom chambers in the Transwell system for invasion. The medium containing 10% FBS (600 μL) were added in the basolateral chamber. After 24 hours, the cells on the surface of the basolateral chamber were fixed with 4% paraformaldehyde for 15 minutes, stained with 0.1% crystal violet for 15 minutes. The cells were photographed in 4 randomly selected visual fields under a microscope.

**Supplementary Figures**

**Supplementary Fig 1. Localization and expression of NSUN2 in CRC cell lines.** A. Immunofluorescence analysis of NSUN2 expression and localization in human normal colorectal mucosal cells (FHC) and CRC cells (HCT116). Scale bar, 20μm. B. Western blot analysis of NSUN2 protein levels in CRC cells.


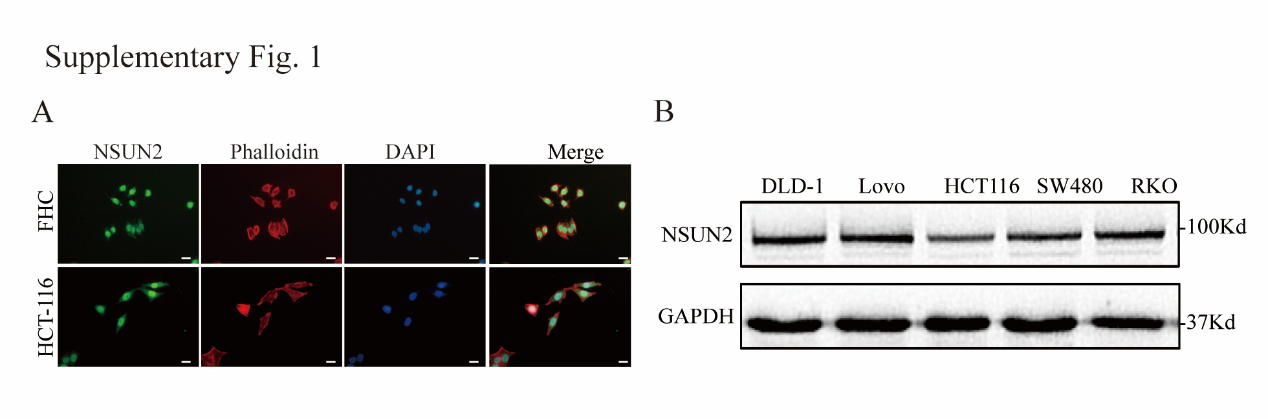


**Supplementary Fig 2. Generation of NSUN2-KO CRC cell lines using the CRISPR/Cas9 system.** HCT116 NSUN2-KO monoclone 3 and DLD-1 NSUN2-KO monoclone 1 were chosen for subsequent experiments.


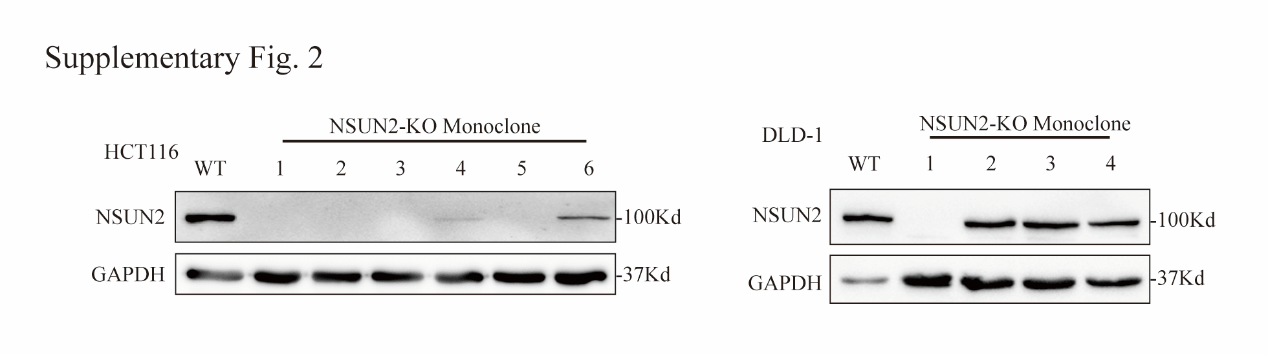


**Supplementary Fig 3. The statistical results of Transwell assays.** The impact of NSUN2-WT and NSUN2-DM rescue on the metastatic capabilities of NSUN2-knockout CRC cells. *P < 0.05, **P < 0.01, and ***P < 0.001.


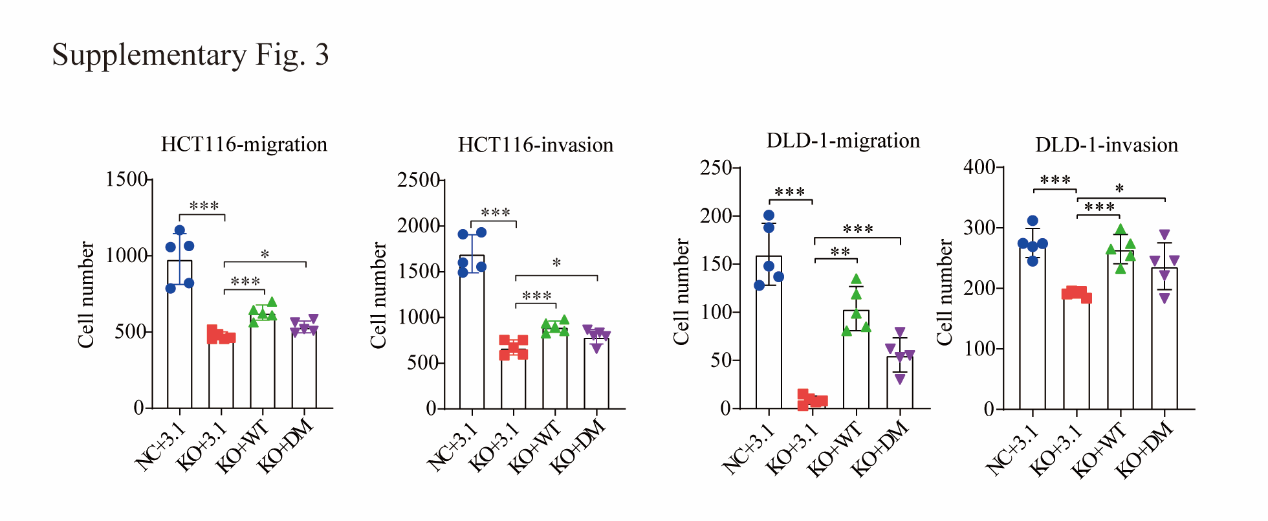


**Supplementary Fig 4. CUL4B knockdown inhibits the proliferation and metastasis of CRC cells.** A. Western blot analysis of CUL4B knockdown efficiency in HCT116 and DLD-1 cells. B. The proliferation ability of CRC cells silencing CUL4B as determined using CCK-8 assay. C. The migration abilities of CRC cells silencing CUL4B as determined using Transwell assays. D. Metastasis of CUL4B-knockdown CRC cells and following NSUN2 (WT and DM) rescue. *P < 0.05, **P < 0.01, and ***P < 0.001.


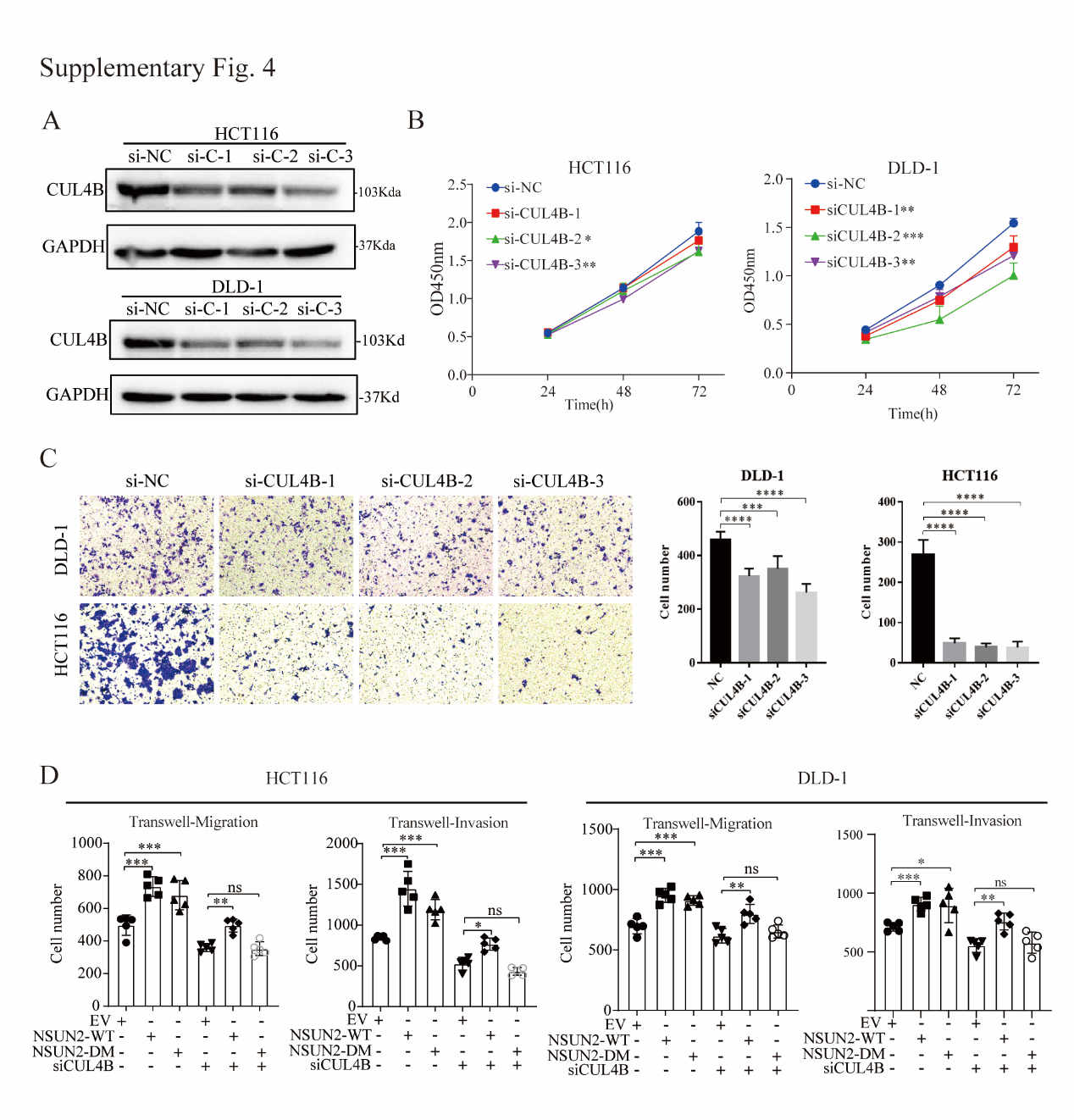


**Supplementary Fig 5. Representative IHC staining images and Co-expression Analysis of NSUN2, CUL4B, EGFR, and HER2 in Xenograft tissues.**


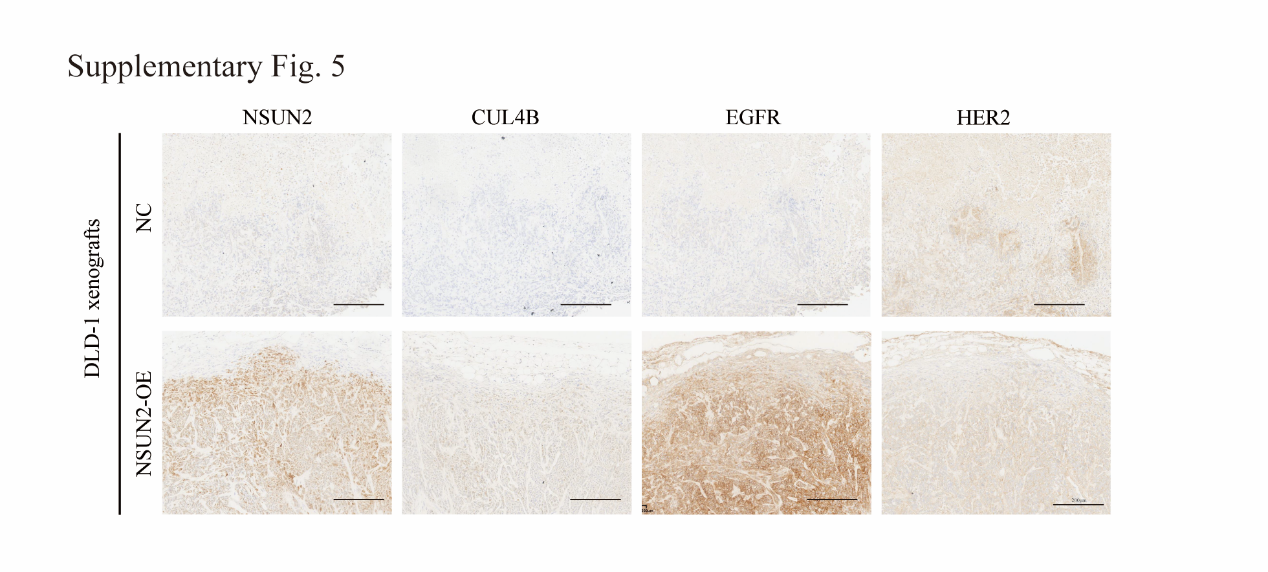


**Table S1. The sequence of small interfering RNA (siRNA) used in this study.**

| **Name** | **Sense (5’-3’)** |
| --- | --- |
| si-CUL4B-1 | GGTGAACACTTAACAGCAA |
| si-CUL4B-2 | CTACCACCGTCTCTAGCTT |
| si-CUL4B-3 | GAAGGAATGTTTAAAGACA |

**Table S2. The antibodies used in this study.**

| **Name** | **Dilution** | **Source** |
| --- | --- | --- |
| NSUN2 | 1:5000 WB  1:800 IHC  1:200 IF | Proteintech, 20854‐1‐AP |
| CUL4B | 1:5000 WB | Proteintech, 12916-1-AP |
| GAPDH | 1:1000 WB | Proteintech, 60004-1-Ig |
| FLAG-Tag | 1:5000 WB | Proteintech, 20543-1-AP |
| HA-Tag | 1:1000 WB | CST, 2376 |
| EGFR | 1:1000 WB | CST, 2085 |
| p-EGFR | 1:1000 WB | CST, 3777 |
| HER2 | 1:1000 WB | CST, 4290 |
| STAT3 | 1:1000 WB | CST, 9139 |
| Phospho-STAT3 (Tyr705) | 1:1000 WB | CST, 9145 |
| Ki-67 | 1:300 IHC | Servicebio, GB111499 |
| Anti-rabbit IgG, HRP-linked Antibody | 1:5000 WB | CST, 7074 |
| Anti-mouse IgG, HRP-linked Antibody | 1:5000 WB | CST, 7076 |
| Goat anti-Rabbit IgG (H+L) Highly Cross-Adsorbed Secondary Antibody, Alexa Fluor™ 488 | 1:1000 IF | Invitrogen, A-11034 |
| Alexa Fluor™ 594 Phalloidin | 1:50 IF | Invitrogen, A12381 |
